# Twice-Weekly Outpatient Rehabilitation Intervention for Young Children With Spinal Muscular Atrophy Treated With Genetic-Based Therapies: Protocol for a Feasibility Study

**DOI:** 10.2196/46363

**Published:** 2023-11-02

**Authors:** Christina Ippolito, Lathushikka Canthiya, Amanda Floreani, Kathleen Luckhart, Andrea Hoffman, Laura McAdam

**Affiliations:** 1 Bloorview Research Institute Holland Bloorview Kids Rehabilitation Hospital Toronto, ON Canada; 2 Holland Bloorview Kids Rehabilitation Hospital Toronto, ON Canada; 3 Department of Paediatrics Temerty Faculty of Medicine University of Toronto Toronto, ON Canada

**Keywords:** active rehabilitation, atrophy, child, feasibility trial, feasibility, genetic-based, genetic-based therapy, infant, occupation therapy, pediatric, physical therapy, physiotherapy, pilot trial, rehabilitation, spinal muscular atrophy

## Abstract

**Background:**

Spinal muscular atrophy (SMA) is a progressive neuromuscular disorder that causes muscle weakness and is the leading genetic cause of infant mortality worldwide. While no definitive cure exists, the approval of 3 genetic-based therapies in Canada since 2018 has led to significant improvements in muscle function for children with SMA. With that, there are no evidence-based rehabilitation interventions and minimal evidence on the combined effects of genetic-based therapies and rehabilitation.

**Objective:**

This protocol describes the methodology to assess the feasibility of a twice-weekly outpatient rehabilitation intervention focusing on gross and fine motor function to inform the methodology and sample size of a definitive clinical trial.

**Methods:**

We will conduct a single-center nonrandomized pilot and feasibility trial to explore an outpatient rehabilitation intervention for children aged 6 months to 3 years with SMA treated with genetic-based therapies. Participation in the study will occur over a 25-week period, with a baseline assessment visit followed by a 12-week intervention period and a 12-week nonintervention period. The rehabilitation intervention comprises weekly physical and occupational therapy for 11 weeks. Assessments will occur at baseline (week 0), end of intervention or early withdrawal (week 12), and follow-up (week 24). Predetermined feasibility indicators will evaluate study feasibility across process (recruitment rates, eligibility criteria, adherence rates, retention rates, questionnaire suitability, and acceptability), resource (time, implementation, and execution), management (materials and data), and scientific (safety, tolerability, and preliminary efficacy) domains.

**Results:**

This project was funded in March 2022, and data will be collected between March 2023 and December 2023. Data analysis will occur between January 2024 and March 2024, with publication expected in the fall of 2024. The protocol for the feasibility trial will be considered successful if it meets the success criteria set out for the feasibility indicators. Indicators of specific interest include all process indicators, as well as time. Exploratory indicators will be reported. Pragmatically, the results of the feasibility trial will inform changes to the protocol and the start-up of a definitive multisite trial.

**Conclusions:**

This novel twice-weekly outpatient rehabilitation intervention will be the first step toward filling the need for an evidence-based rehabilitation intervention for children with SMA treated with genetic-based therapies. It is expected that consistent and intensive rehabilitation therapy will augment functional gains being observed in this population. In the future, a definitive trial will measure the efficacy of the intervention.

**Trial Registration:**

ClinicalTrials.gov NCT05638750; https://clinicaltrials.gov/study/NCT05638750

**International Registered Report Identifier (IRRID):**

DERR1-10.2196/46363

## Introduction

Spinal muscular atrophy (SMA) is a progressive neuromuscular disorder in which there is a gradual degeneration of motor neurons due to a loss-of-function mutation in the survival of motor neuron 1 (SMN1) gene, which enables proper functioning of motor neurons and therefore muscle function [1-3]. There are 3 pediatric subtypes (types I, II, and III) of SMA based on the age of onset of muscle weakness and motor milestones achieved [3]. All SMA subtypes demonstrate a decline in function over time. SMA type I presents the most rapid onset and severe decline [1,4,5]. SMA types II and III have a later onset and more gradual decline [5,6]. SMA affects 1 in 6000 to 10,000 births worldwide [7-10]. As of 2020 in Canada, there are an estimated 700 to 2140 cases of SMA [11,12] with approximately 35 new cases per year [10]. SMA is one of the most common genetic causes of mortality in infants [3,13]; however, there is optimism that this will change due to the availability of new treatments.

Health Canada has approved 3 genetic-based therapies for individuals with SMA: nusinersen (brand name Spinraza, 2018), onasemnogene abeparvovec (brand name Zolgensma, 2020), and risdiplam (brand name Evrysdi, 2021). These genetic-based therapies aim to increase the production of the SMN protein and improve muscle function [14]. As a result of these new treatments, Ontario, Canada, has included SMA in newborn screening programs since 2020, enabling treatment shortly after birth [15]. Implementation of these genetic-based therapies has demonstrated significant and clinically meaningful gains in physical function in clinical trials in both individuals receiving a genetic-based therapy presymptomatically as well as after the onset of clinical symptoms, which was previously unheard of in the SMA population [16-24]. For example, presymptomatic infants who received nusinersen within 6 weeks of birth gained the ability to sit without support (100%), walk with assistance (92%), and walk independently (88%) [18]. Additionally, 57% of children with later-onset SMA treated with nusinersen saw increases in motor function compared to 26% of children in the placebo control group [22].

The most recent standards of care for SMA were published in 2018, before the widespread approval of genetic-based treatments internationally [14,22,25]. Additionally, there is minimal evidence-based guidance in the literature regarding rehabilitation interventions to proactively optimize function for children with SMA treated with genetic-based therapies [3]. With that, there is a paucity of published long-term evidence regarding rehabilitation programs and their impact on physical function in individuals with SMA treated with genetic-based therapies to guide program development and funding of rehabilitation programs. Few studies have explored the combined effects of genetic-based therapy and rehabilitation therapy for children with SMA; however, preliminary research indicates promising synergies [26]. For example, a retrospective study reported that individuals treated with nusinersen who attended physiotherapy (PT) 5 days per week experienced a 12.7% (*P*<.001) improvement in gross motor gains compared to a 5.4% increase in those who only received nusinersen and less than 1 PT session per week at 1 year after starting nusinersen [26].

Due to the progression of SMA, PT and occupational therapy (OT) historically focused on the provision of equipment, compensatory strategies, and reducing the risk of secondary complications, such as joint contractures [3]. With new disease-modifying treatments, families of young children with SMA are seeking proactive rehabilitation to enhance and optimize physical function [27,28]. The intervention presented in this protocol proposes rehabilitation therapy that draws upon models of service delivery used in pediatric populations at the delivering institution. Goal-directed therapy will be implemented, which has been shown to be the most effective type of therapy for commonly studied pediatric neurological conditions such as cerebral palsy [29-31]. Additionally, based on previous SMA exercise therapy intervention studies that were tolerable and safe [32], a 12-week therapy block duration has been proposed. Drawing from the aforementioned proposal, further evaluation of the effects of therapy and the efficacy of this rehabilitation program is crucial to improving the quality of care for this population and the optimization of their function [26].

The objective of the study is to assess the feasibility of a twice-weekly outpatient rehabilitation intervention focused on gross motor and manual function to inform the methodology and sample size of a definitive clinical trial according to specific feasibility indicators (process, resources, management, and scientific) [33]. This protocol will describe the pilot and feasibility trial for this evidence-informed outpatient rehabilitation intervention for children with SMA treated with genetic-based therapies. The Standard Protocol Items–Recommendations for Interventional Trials (SPIRIT) guidelines, the Consolidated Standards of Reporting Trials (CONSORT) extension for feasibility and pilot trials, and the Template for Intervention Description and Replication (TIDieR) checklist for intervention description and replication guided the reporting of this protocol.

## Methods

### Design

The study is a single-center nonrandomized pilot and feasibility trial to explore the feasibility of an outpatient rehabilitation intervention for children 6 months to 3 years of age with SMA treated with genetic-based therapies. Participation in the study will occur over a 25-week period, with a baseline assessment visit followed by a 12-week intervention period and a 12-week nonintervention period. The trial site will be a pediatric rehabilitation hospital in Toronto, Canada.

### Participants

A convenience sample of 5-10 participants (children and parent or guardian dyads) will be enrolled. Current standards of practice deem formal sample size calculations unnecessary for feasibility trials since findings obtained from the small sample size in this study will allow for generalization to a larger definitive trial. Thus, a pragmatic approach will be undertaken, justifying the sample size used in this study [34]. This also supports the practicality of this study design, as the participant pool does not need to be exhausted to deliver the intervention.

### Eligibility Criteria

The inclusion criteria are as follows: (1) SMA (type I, II, or III) diagnosis; (2) received or receiving a genetic-based therapy; (3) age 6 months to 3 years; (4) able to participate in twice-weekly therapy at the study site; (5) able to bring appropriate respiratory equipment to weekly therapy sessions, if required; (6) parent or guardian must be able to speak and read English; (7) child participant must be able to understand and follow directions in English, as age appropriate; and (8) parent or guardian must consent to participate on behalf of their child.

The exclusion criteria are as follows: (1) live outside of Ontario, and (2) tracheostomy or use of daytime ventilation (excluding ventilation used during naps).

Inclusion and exclusion criteria will be assessed during the recruitment process. Respiratory safety screening will be conducted by the principal investigator if the use of respiratory equipment is identified.

### Recruitment

**Figure 1:**
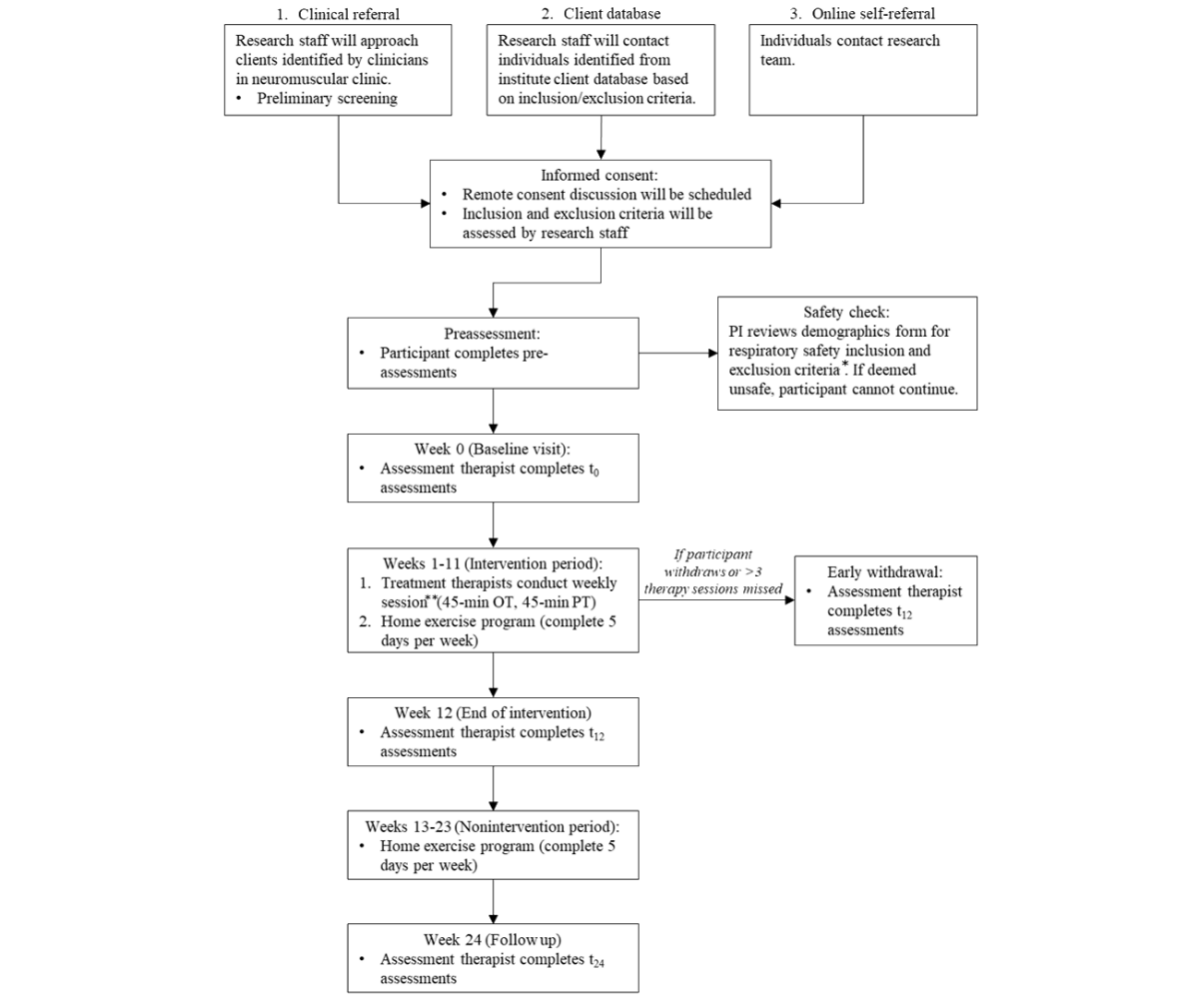
Recruitment path and participant flow for the twice-weekly outpatient intensive rehabilitation intervention for young children with spinal muscular atrophy treated with genetic-based therapies. *The respiratory safety inclusion and exclusion criteria include 5 inclusion criteria and 2 exclusion criteria. **Up to 3 weeks of therapy sessions can be missed or held on the internet (ie, 3 OT and 3 PT sessions may be missed or held virtually). OT: occupational therapy; PI: principal investigator; PT: physiotherapy.

Participants will be recruited from across Ontario, Canada. A multipronged recruitment strategy will be implemented using active (clinical referrals and an institutional research client database) and passive (self-referral through web posts) approaches. Posts include institutional internet posts (social media and the institution’s website) as well as social media sharing on websites commonly accessed by families with children with SMA. Figure 1 describes potential recruitment pathways and participant flow.

### Intervention

Parents will consent on behalf of their child before study visits are conducted by trained research staff. The intervention comprises a 12-week intervention period (OT and PT sessions plus a home exercise program) followed by a 12-week nonintervention period (home exercise program only). The 12-week intervention period was based on previous exercise therapy studies on pediatric neurological conditions such as cerebral palsy [29-31], and a small number of SMA exercise studies [23,32,35,36]. The on-off block structure is what is typically offered at the institution.

Participants will not be required to stop any additional private therapies during the study. However, participants will be asked not to start any new treatments or therapy during the study. All additional activities should be recorded in the home exercise diary.

In week 0, therapy goals will be set using the Canadian Occupational Performance Measure (COPM) and Goal Attainment Scaling (GAS) goals created with the participant’s parents, and baseline functional assessments will be completed. OT and PT goals will be individual to each participant. With that, the therapy sessions are created and personalized to the participant and their goals.

During the intervention period, active therapy will be delivered in person through weeks 1-11. A total of 2 therapy sessions (one 45-minute PT and one 45-minute OT session) per week in addition to a home exercise program will be completed. It will be recommended that participants complete the home exercise program 5 days per week in addition to the 2 therapy sessions (OT and PT). During the nonintervention period, the home exercise program will be continued. Functional assessments will be reevaluated at week 12 and then again at week 24 to determine whether functional gains were maintained. See Figure 1 for a detailed participant flow. A total of 3 weeks of sessions will be permitted to be missed or conducted through video conferencing software over the intervention period.

During the nonintervention period, the participant will receive an updated home exercise program to be completed 5 days per week. A research team member will contact the participant every 2 weeks to review the home exercise diary and other activities in which the participant is participating and to reiterate the importance of the home exercise program.

All on-site therapy and assessment sessions will be completed in a private therapy room; however, sessions could occur in a group therapy room if needed.

### Assessment

Assessments will be administered at prescribed time points, as described in Table 1.

**Table 1:** Intervention and assessment time points for the twice-weekly outpatient rehabilitation intervention for young children with spinal muscular atrophy treated with genetic-based therapies. The intervention period occurs between *t*_1_ and *t*_11_, and the nonintervention period occurs between *t*_13_ and *t*_23_.

Measure	Assessment			
	Preassessment	*t*_0_ (baseline)	*t*_12_ and *t*_10_ (EOI^a^ and EW^b^)	*t*_24_ (follow-up)
Demographics survey^c^	✓			
ITQOL^c,d^		✓	✓	✓
COPM^e,f^		✓		
GAS^e,g^		✓		
CHOP-INTEND^h^ or HFMSE^e,i,j^		✓	✓	✓
Bayley (All)^e^		✓		
Bayley (motor domain only)^e^			✓	✓
Evaluate COPM and GAS goals^e^			✓	✓
AIM^k^, IAM^l^, FIM^c,m^			✓	

^a^EOI: end of intervention.

^b^EW: early withdrawal.

^c^Completed by participant parent or guardian.

^d^ITQOL: Infant Toddler Quality of Life Questionnaire.

^e^Conducted by assessment therapist.

^f^COPM: Canadian Occupational Performance Measure.

^g^GAS: Goal Attainment Scaling.

^h^CHOP-INTEND: Children’s Hospital of Philadelphia Infant Test of Neuromuscular Disorders.

^i^CHOP-INTEND used with nonindependent sitters and HFMSE used with independent sitters.

^j^HFMSE: Hammersmith Functional Motor Scale-Expanded.

^k^AIM: Acceptability of Intervention Measure.

^l^IAM: Intervention Appropriateness Measure.

^m^FIM: Feasibility of Intervention Measure.

### Intervention Providers

The intervention uses 2 types of providers: assessment therapists and treatment therapists. Assessment therapists will complete the listed outcome measures at week 0, end of intervention or early withdrawal (week 12), and follow-up (week 24). Treatment therapists will plan and conduct the therapy sessions based on the COPM and GAS goals created by the assessment therapists. For a participant, the assessment and treatment therapists will be different to avoid bias.

The assessment and treatment therapists will be OTs and PTs with previous experience and training working with young children with SMA. Assessment therapists will complete the standardized training for the respective assessments. Assessment OTs will complete the Bayley Scale (all sections except the gross motor function), COPM, and GAS. Assessment PTs will complete the Children’s Hospital of Philadelphia Infant Test of Neuromuscular Disorders (CHOP-INTEND) or Hammersmith Functional Motor Scale-Expanded (HFMSE), gross motor section of the Bayley Scale, COPM, and GAS.

### Outcome Measures

#### Demographics Survey

This 15-item purposefully developed survey will capture participant characteristics (eg, age at intervention commencement [year and month], sex, parent report of genetic condition, therapies received, and timing or frequency).

#### Infant Toddler Quality of Life Questionnaire

The ITQOL (Infant Toddler Quality of Life Questionnaire) [37] comprises 103 items on 12 scales pertaining to the past 4 weeks, covering physical and psychosocial domains and the impact of child health on parents. Scales include physical functioning, growth and development, bodily pain, temperament or moods, general behavior, getting along, general health perception, parental-emotional, parental time, family activities, family cohesion, and change in health. Multi-item scales showed adequate internal consistency (Cronbach α=.81). Overall, 4 scales showed adequate (intraclass correlation; ICC ≥0.70; *P*<.01), 6 scales showed moderate (ICC=0.50-0.70; *P*<.01), and 1 scale had poor test-retest reliability [37].

#### COPM Measure

The COPM [38,39] measures an individual’s self-perception of occupational performance on self-care, productivity, and leisure and is used by OTs in initial assessments to set goals and plan treatment focusing on activities that an individual needs, wants, or is expected to do. The importance of activities is rated on a 10-point scale (1=not important at all to 10=extremely important). The individual selects the 5 most important activities, which are rated on a 10-point performance scale (1=not at all able to 10=able to perform extremely well) and for satisfaction (1=not at all satisfied to 10=extremely satisfied).

#### GAS Measure

GAS [40,41] will evaluate an individual’s progress toward participant and family goals. Each scale is developed based on assessment and collaboration between the therapist and the participant and family. A 5-point scale, ranging from –2 to +2, is used. A numeric value is assigned to each level of performance. On the scale, 0 is used to represent the expected level of outcome, with +1 and +2 indicating greater than expected progress and –1 and –2 indicating less than expected progress.

#### CHOP-INTEND Measure

The CHOP-INTEND [42] evaluates the motor skills of individuals with SMA type I aged 3 months to 4 years or older over 16 items. Items are scored on a 4-point scale (0=no response, 1=partial level of response, 3=nearly full level of response, 4=complete level of response). The assessment has good internal consistency (Cronbach α>.70) and strong intrarater reliability (ICC=0.96). CHOP-INTEND will be used with participants who do not sit independently.

#### HFMSE Measure

The 33-item HFMSE [43] assesses the functional motor ability of individuals with SMA who are able to sit and walk using a 3-point scoring system (0=unable to perform, 1=performs with modification, adaptation, and compensation, 2=performs without modification, adaptation, and compensation). The HFMSE will be used with participants who are able to sit independently.

#### Bayley Scales of Infant and Toddler Development, 4th Edition

The Bayley Scale is a developmental assessment tool for diagnosing developmental delays in early childhood (16 days to 42 months) across 5 domains: cognitive, language, motor, social-emotional, and adaptive behavior. The entire Bayley Scale can take 30-70 minutes to administer, depending on the participant’s age. The entire Bayley Scale will be completed at week 0, and only the motor scales will be completed at weeks 12 and 24.

#### Acceptability of Intervention Measure, Intervention Appropriateness Measure, Feasibility of Intervention Measure

The Acceptability of Intervention Measure (AIM), Intervention Appropriateness Measure (IAM), and Feasibility of Intervention Measure (FIM) [44] are each 4-item surveys to evaluate the acceptability, appropriateness, and feasibility of the intervention from a personal perspective using a 5-point Likert scale (1=completely disagree to 5=completely agree). Internal consistency (α) of the 3 measures ranged from .87 to .89, and they take less than 5 minutes each to complete.

#### Home Exercise Diary

This purposefully developed form will be completed by the participant’s parent or guardian each time the home exercise program is completed. During the intervention period, the diary will be reviewed by the therapists weekly, and during the nonintervention period, a research team member will call the participant every 2 weeks to review the diary. For each time the home exercise program is completed, the participant will complete the intervention week, the day of the week (eg, Monday), OT and PT exercises completed, and any comments (ie, about the participant’s mood, adverse events, and duration of the exercises). Additionally, the parent or guardian will indicate if any additional activities were completed.

### Data Management

All assessment measures except the participant-facing measures (ie, demographics survey, ITQOL, AIM, IAM, and FIM) will be completed in hard copy. A trained research team member will input the data into Research Electronic Data Capture (REDCap) [45,46] forms for secure retention and ease of exportation and analysis. Participant-facing measures will be completed by parents or guardians electronically on REDCap.

### Feasibility Indicators and Data Analysis

The trial’s feasibility will be evaluated using process, resource, management, and scientific domains [33]. Specific feasibility indicators within the domains, their data management plan, and success criteria are described in Table 2.

Process indicators will examine key items to ensure the success of the trial. Recruitment rate, eligibility criteria, adherence rate, and retention rate data will be collected from recruitment logs. Results for these indicators will be reported as ratios, means, and frequencies. Questionnaire completion times and completion correctness will be reported as means and frequencies. Item scores for acceptability measures (AIM, FIM, and IAM) will be summed by each participant and reported as averages for each measure. Resource indicators will assess time and resource issues that could occur when running a definitive trial. Therapists will record session and documentation durations for all visits. These results will be presented as a ratio and compared to the success criteria. With the same data, session and documentation times will be compared to similar values in the literature as well as clinician reports. Last, protocol deviations will be reviewed by deductive content analysis to determine if protocol revisions are required. Management indicators will assess potential human and data management issues when running the trial and will be evaluated through deductive content analysis of completed measures and data analysis notes. Adverse events will be analyzed by deductive content analysis. Feasibility indicators will be compared to the success criteria, as appropriate.

From a scientific standpoint, a preliminary examination of the intervention’s effectiveness will be assessed through safety and efficacy. Participant demographics and medical history collected from the demographics survey will be summarized descriptively. Preliminary efficacy will be evaluated through descriptive statistics and within-subject change scores of intervention assessment measures (ie, ITQOL, CHOP-INTEND or HFMSE, Bayley, COPM, and GAS goals). No between-subject analysis will be completed due to the small sample size. Change scores will measure functional gains and losses during the intervention period (*t*_12_-*t*_0_), nonintervention period (*t*_24_-*t*_12_), and the entire trial (*t*_24_-*t*_0_) for each participant separately.

**Table 2:** Feasibility indicators to measure the success of the twice-weekly outpatient intensive rehabilitation intervention for young children with spinal muscular atrophy treated with genetic-based therapies with data management (data collection methods and analysis) and a priori success criteria.

Feasibility indicator		Data management	Success criteria
**Process**			
	Recruitment rates	Ratio of enrolled participants vs approached potential participants; number screened and enrolled per month; and time from screening to enrollment to baseline visit	≥10% response rate from eligible participants [47]
	Eligibility criteria	Ratio of eligible vs screened participants	>70% meet eligibility criteria
	Adherence rates	Therapist documentation (attendance at study visits) Home exercise diary during intervention and nonintervention (weekly average completion rate of prescribed home exercise program)	≥75% attendance to therapy sessions^a^ At least three days per week
	Retention rates	Ratio of participants who successfully complete the study (including outcome measures) vs enrolled participants	≥70% retention of participants [48]
	Questionnaires	Participant-reported outcome measures (time to complete forms and correctly completed forms)	Exploratory
	Acceptability	AIM^b^, IAM^c^, and FIM^d^	≥90% on the AIM, IAM, and FIM [49]
**Resources**			
	Time	Therapist documentation (ratio of actual vs estimated durations)	Assessment: meets time prescribed in protocol (±5 min) Documentation: ≤15 min
	Implementation	Therapist documentation (cost analysis of therapist time compared to standard care)	Exploratory
	Execution	Protocol deviations (amount, frequency, and type)	Exploratory
**Management**			
	Materials	Patient-facing materials and outcome measures (evaluate whether revisions are required)	Exploratory
	Data	Data analysis notes (evaluate if additional data are required for efficacy analysis in a definitive trial)	Exploratory
**Scientific**			
	Safety and tolerability	AE^e^ and SAE^f^ documentation (type and cause)	Exploratory
	Preliminary efficacy	Efficacy (functional assessment change scores between *t*_0_, *t*_12_, and *t*_24_ time points for within-subject changes) Sample size calculations for a larger definitive trial	N/A^g^

^a^A total of 3 weeks of sessions will be permitted to be missed or conducted through video conferencing software over the course of the 12-week intervention period.

^b^AIM: Acceptability of Intervention Measure.

^c^IAM: Intervention Appropriateness Measure.

^d^FIM: Feasibility of Intervention Measure.

^e^AE: adverse event.

^f^SAE: serious adverse event.

^g^N/A: not applicable.

The sample size for a larger equivalence trial will be calculated [50]. Specifically, the trial will test whether the intervention (genetic-based therapy and the intervention) and standard treatment (genetic-based therapy only) are equally effective. With that, there are 2 null hypotheses: 
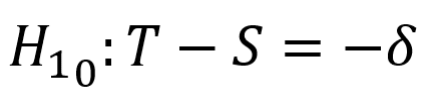
 and 
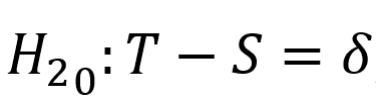
, where T=intervention, S=standard treatment, and *δ*=clinically admissible margin of equivalence. Using the information gained from the completed outcome measures, a primary end point will be confirmed. Calculations for continuous outcome measures by Zhong will be used:



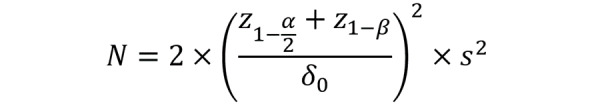



Where *N*=size per group; *z_x_*=standard normal deviation for 1- or 2-sided 
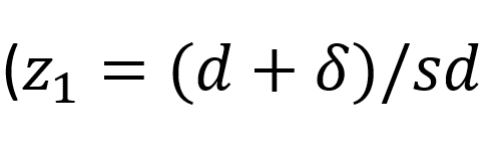
 and 
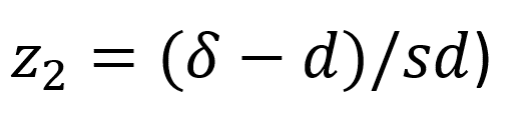
; *δ*_0_=clinically acceptable margin; and *s*^2^=polled SD of both comparison groups. As per convention, α will be set at .05 and β will be set at .80. *δ*_0_ will be calculated as the difference between the mean changes in the chosen primary end point for the current intervention (taken from this feasibility study) and typical change scores for the age-matched populations from the literature minus one.

### Monitoring

A data safety monitoring board (DSMB) will be established to review and evaluate the accumulated study data for participant safety. The DSMB will function independently from the study investigators and have no direct involvement with study conduct. Any recommendations regarding the continuation, modification, or termination of the trial will be made to the study investigators based on the received reviews. A DSMB term of reference was prepared and is available upon request.

Adverse events or serious adverse events will be collected and reported by therapists and research personnel. The study doctors will assess the seriousness of adverse events or other unintended effects and will propose a management plan as required. Serious adverse events will be reported to the DSMB.

### Ethical Considerations

This study has been approved by the Research Ethics Board at Holland Bloorview Kids Rehabilitation Hospital in Toronto, Canada (REB#0550). All participants, through a substitute decision-maker, will partake in an informed consent discussion and sign an informed consent form before being enrolled in this study.

## Results

This project was funded in March 2022, and data will be collected between March 2023 and December 2023. Data analysis will occur between January 2024 and March 2024, with publication expected in the fall of 2024.

This feasibility trial will be considered successful if it meets the success criteria set for the feasibility indicators. Indicators of specific interest include all process indicators as well as time. Exploratory indicators will be reported. Pragmatically, the results of the feasibility trial will inform updates to the design of the intervention and the protocol, as well as the start-up of a definitive multisite trial.

## Discussion

This nonrandomized pilot and feasibility trial aims to assess the feasibility of the twice-weekly outpatient rehabilitation intervention for young children with SMA treated with genetic-based therapies. The description of this protocol and the future results are poised to make several valuable contributions to the functional and motor capabilities of children with SMA.

First, this innovative study is one of the first of its kind and will provide insight into the therapy potential for young children with SMA treated with genetic-based therapies. With the approval of genetic-based therapies to treat individuals with SMA as well as the introduction of newborn screening across Canada within the past 5 years, children are being diagnosed and treated earlier in the disease course [15]. With these previously unheard-of functional opportunities and the lack of knowledge of the long-term efficacy and tolerability of genetic-based therapies, it is important for children with SMA treated with genetic-based therapies to receive appropriate rehabilitation therapy. The current SMA standards of care include recommendations for exercise therapy as well as PT and OT involvement in bracing, positioning, and equipment [3,25]. However, these recommendations were published before the widespread approval of genetic-based therapies, and therefore the efficacy of PT and OT treatment blocks in this population has yet to be explored. This protocol will be a first step toward establishing an evidence-based rehabilitation intervention therapy program for children with SMA treated with genetic-based therapies.

Additionally, previous studies and therapy recommendations for children with SMA identify therapies to maintain function [23,32,35,36,51]. However, the proposed outpatient rehabilitation intervention is hypothesized to increase function in participants in combination with genetic-based therapies.

While there are several promising outcomes to the proposed pilot and feasibility trial, it does have its limitations. For instance, the implementation of the nonrandomized study design will not yield definitive efficacy data at this time. Additionally, the nonrandomized study design will not enable the measurement of subgroup differences. With that, nonrandomized pilot studies are still an effective method to evaluate a novel intervention, such as the trial outlined in this protocol [52]. It is expected that this pilot and feasibility trial will provide important insight into both the feasibility of the intervention itself and a future definitive trial.

Challenges to the implementation and feasibility of the intervention are foreseen. Recruitment will be a challenge due to the rarity of the disease and the large recruitment area (ie, anywhere in the province of Ontario, Canada). However, it is expected that families will travel far distances to receive therapy sessions at no cost with therapists with expertise working with young children with SMA. Additionally, the long potential travel distances may impact retention, adherence, and protocol deviations. However, the protocol includes that 3 OT and PT sessions can be held on video conferencing software or be missed. This allows families and therapists to be flexible in the delivery of the sessions without encountering large amounts of protocol deviation. Last, adherence to the home exercise program, particularly during the nonintervention period, may be a challenge. In past clinical experiences, the clinical team has seen functional gains lost when home exercises were not completed after a therapy block. Therefore, a research team member will contact the family every 2 weeks during the nonintervention period to remind them of the importance of completing the home exercise program.

Overall, it is expected that when coupled with available genetic-based therapies, the consistent PT and OT provided as part of the proposed rehabilitation intervention will augment the functional gains being observed in this treated population. We aim for this feasibility trial to reflect the realities of participants and their families, as well as therapists and institutions that may deliver this intervention. In sharing this protocol, we hope to achieve widespread awareness of this new potential rehabilitation intervention with the future goal of making direct clinical impacts in the SMA community.

## Abbreviations

AIM: Acceptability of Intervention Measure
CHOP-INTEND: Children’s Hospital of Philadelphia Infant Test of Neuromuscular Disorders
CONSORT: Consolidated Standards of Reporting Trials
COPM: Canadian Occupational Performance Measure
DSMB: data safety monitoring board
FIM: Feasibility of Intervention Measure
GAS: Goal Attainment Scaling
HFMSE: Hammersmith Functional Motor Scale-Expanded
IAM: Intervention Appropriateness Measure
ICC: intraclass correlation
ITQOL: Infant Toddler Quality of Life Questionnaire
OT: occupational therapy
PT: physiotherapy
REDCap: Research Electronic Data Capture
SMA: spinal muscular atrophy
SMN1: survival of motor neuron 1
SPIRIT: Standard Protocol Items—Recommendations for Interventional Trials
TIDieR: Template for Intervention Description and Replication

## Data Availability

The data sets generated during and/or analyzed during this study are available from the corresponding author on reasonable request. Further details can be provided by the authors to interested parties when the request is made.
